# From Bioinspired Topographies toward Non-Wettable Neural Implants

**DOI:** 10.3390/mi14101846

**Published:** 2023-09-27

**Authors:** Ali Sharbatian, Kalyani Devkota, Danesh Ashouri Vajari, Thomas Stieglitz

**Affiliations:** 1 Laboratory for Biomedical Microtechnology, Department of Microsystems Engineering (IMTEK), University of Freiburg, 79110 Freiburg, Germany; ali.sharbatian@imtek.uni-freiburg.de (A.S.); thomas.stieglitz@imtek.uni-freiburg.de (T.S.); 2 BrainLinks BrainTools, Institute for Machine-Brain Interfacing Technology (IMBIT), University of Freiburg, 79110 Freiburg, Germany; 3 Bernstein Center Freiburg, University of Freiburg, 79104 Freiburg, Germany

**Keywords:** non-wettability, microcavity, micropillars, air-pocket, overhang layer

## Abstract

The present study investigates different design strategies to produce non-wettable micropatterned surfaces. In addition to the classical method of measuring the contact angle, the non-wettability is also discussed by means of the immersion test. Inspired by non-wettable structures found in nature, the effects of features such as reentrant cavities, micropillars, and overhanging layers are studied. We show that a densely populated array of small diameter cavities exhibits superior non-wettability, with 65% of the cavities remaining intact after 24 h of full immersion in water. In addition, it is suggested that the wetting transition time is influenced by the length of the overhanging layer as well as by the number of columns within the cavity. Our findings indicate a non-wetting performance that is three times longer than previously reported in the literature for a small, densely populated design with cavities as small as 10 μm in diameter. Such properties are particularly beneficial for neural implants as they may reduce the interface between the body fluid and the solid state, thereby minimiing the inflammatory response following implantation injury. In order to assess the effectiveness of this approach in reducing the immune response induced by neural implants, further in vitro and in vivo studies will be essential.

## 1. Introduction

The wetting process can be found in a wide range of everyday biological and industrial systems. Contact angle (CA) is a commonly used quantitative reference for the wettability of a surface, which measures typically greater than 0° and less than 180° [1]. Despite being the most commonly used method to assess surface non-wettability, conclusions about intrinsically wetting materials based on contact angle measurements alone can be misleading. The immersion test is a simple and yet more appropriate method for assessing wettability. In this method, the sample is completely immersed in the test liquid and the degassing is monitored over a period of time. Full immersion provides an additional criterion to the contact angle, helping to better evaluate sample non-wettability [2].

In the context of neural engineering and neural implants, the wetting characteristics of an implant can be of interest as they may have an impact on the initial foreign body response. A foreign body reaction (FBR) in the brain is triggered when biomaterials are implanted in the brain. Protein adsorption, acute inflammation, chronic inflammation, and scar formation around biomaterials are the four phases of foreign body reaction. Immediately after the insertion of a device into the brain, proteins from the blood are adsorbed onto the biomaterial and activate an area of injury [3,4]. In the second stage of an FBR, the acute phase represents the most critical event, which is characterized by a series of pathological reactions triggered by blood–brain barrier (BBB) dysfunction and glial activation [3,5]. This is followed by the chronic inflammatory process, which usually takes place within two to ten days after implantation. It is characterized by changes in the macrophage and macroglial phenotype and the release of cytokines [5]. It has been shown that glial scarring occurs approximately 2–3 weeks after they become activated, leading to encapsulation of the biomaterial by astrocytes and microglia [5,6].

An FBR is caused by nonspecific absorption on the surface of the implant, which affects the capsule formation and may result in implant fouling [6]. Given the critical role that protein adsorption plays in the host tissue response, reducing serum protein adsorption on the implant surface may reduce microglial and macrophage aggregation and activation, thereby further minimizing the inflammatory cascade and neuronal loss [7]. A low fouling interface reduces the adsorption of proteins to its surface, which can affect the first step in the FBR [8]. In this context, the anti-biofouling properties of non-wettable surfaces may be of great interest as they dramatically reduce the surface contact between liquids and solids without the need for additional coatings [9,10]. Such non-wettable properties are also found in nature. Examples include the superhydrophobicity of lotus leaves, the underwater superoleophobicity of fish scales, and the slipperiness of pitcher plants [11,12,13].

The motivation for this work is the use of surface topography for the design of non-wettable surfaces, which can be relevant for neural implants. To this end, we focused on three main geometric aspects of microscopic pattern structures, overhangs, pillars, and feature density, and their influence on the wetting properties. The outcome of this work should provide guidance on different patterning strategies, such as pillar density and feature size adjustments, that can be used to engineer the immune response induced by a neural implant. This work focuses primarily on the surface design and non-wettability performance of the designed structures in vitro. While this study does not discuss the non-wettability in the presence of cells or living tissue, future evaluations should address the interaction between cells and body fluids.

## 2. Materials and Methods

**Figure 1:**
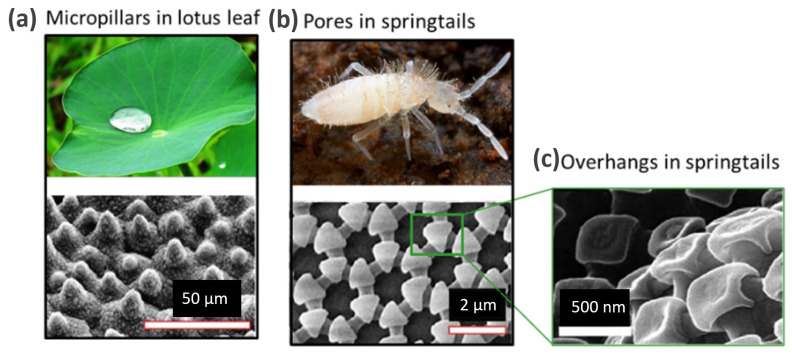
Non-wetting surfaces found in nature and schematic representation of the proposed micropatterns: (**a**) lotus leaf with a related SEM image that demonstrates the presence of pillar-like structures for non-wettability. (**b**) A springtail image with a corresponding SEM image of its skin surface [14], (**c**) a magnified view of (**b**) showing granules with overhanging structures for enhanced non-wettability [14,15]. (Copyright © 2011, Ensikat et al., 2013, American Chemical Society).

In recent decades, bioinspired patterns have been increasingly used to produce functional surfaces for a variety of applications [14,15,16,17]. Many naturally occurring surfaces, such as the lotus leaf and springtails, feature micron-scale patterns showing omniphobic behavior [15], which is defined by the ability to retain air pockets within cavities upon immersion in water [18]. The hydrophobic nature of the lotus leaf surface is due to the presence of such micron-sized pillar-like structures (Figure 1a) [14]. It has also been observed that the surface of the springtail contains nanoscopic granules and interconnecting ridges that form a comb-like pattern (Figure 1b), which imparts omniphobic properties to skin-breathing anthropods [18]. When granules are examined closely (Figure 1c), it becomes apparent that they have an overhang layer; they are shown for their ability to meet the omniphobic characteristics of surfaces [15]. This surface exhibits omniphobicity regardless of the type of liquid it is immersed in, polar or non-polar [18]. Taking into account the above-mentioned structures found in nature, the proposed surface designs focused on the following aspects:

1. The impact of changing the feature density of the air-filled cavity on wettability.
2. The influence of the overhang layer on wettability.
3. The effect of the presence of pillars inside the cavity on wettability.

The proposed designs and respective arrays were fabricated via direct laser writing, and their wettability behavior was evaluated by means of contact angle measurement and immersion testing. 

### 2.1. Design Considerations

**Figure 2:**
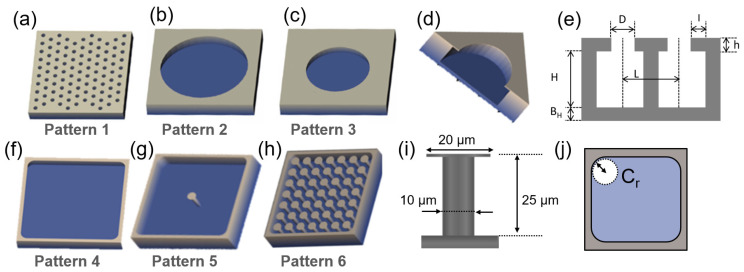
CAD presentation of proposed designs for pattern 1 to pattern 6, respectively; (**a**) pattern 1, consisting of highly populated cavities (opening diameter 10 µm); (**b**,**c**) patterns 2 and 3 featuring single circular cavities with different opening diameters, 190 µm and 160 µm, respectively; (**d**) a cross-sectional view highlighting the overhang layer; (**e**) schematic cross-sectional view of two reentrant cavities next to each other. This view does not show the pillar in the center of the cavity with dimensions specified in Table 1; (**f**–**h**), presenting patterns 4–6, addressing the impacts of pillars in design where the pillar population is increased from no pillar (pattern 4) to 49 pillars (pattern 6); (**i**) cross-sectional view of the pillar in patterns 5 and 6; (**j**) schematic from top view of patterns 4–6, indicating corner curvature radius (Cr); the total printing area of each pattern structure was measured to be 233 μm × 233 μm.

Six designs were considered to investigate the effect of feature density, overhanging layers, and the presence of pillars on the wettability of test surfaces (Figure 2). All the models were designed using AutoCAD (Autodesk 2022, San Rafael, CA, USA). To study the effect of feature density, the wettability of a single large cavity was investigated. This was then compared to the wettability of distributed small air-filled cavities of similar volume. The largest cavity area was limited to 233 µm × 233 µm [10].

**Pattern 1** (P1)—This pattern (P1) consists of 100 cavities with a diameter of 10 µm (see Figure 2f, Table 1).

**Pattern 2** (P2) consists of one single cavity with an opening diameter of 190 µm (Figure 2f, Table 1). It was designed so that P1 and P2 had the same total cavity volume (Figure 2e, Table 1). To investigate the effect of the overhanging layers on the wettability, two designs with different lengths of the overhanging layers were considered.

**Pattern 3** (P3) is an extension of pattern 2, with the overhanging layer enlarged four times (Figure 2f, Table 1). To understand the effect of the presence of pillars within cavities on wettability, three different design variations were considered: without pillars, with single pillars, and with multiple pillars. 

**Pattern 4** (P4) consists of a cavity with an upper curved corner of 233 µm × 233 µm and the radius of the curved corners of 15 µm (Figure 2f, Table 1).

**Pattern 5** (P5) is a modified version of P4 with the addition of a single pillar at its center. The pillar introduced in this variation is 25 µm high and 10 µm wide, with an overhanging diameter of 20 µm (Figure 2f, Table 1).

**Pattern 6** (P6): There are 49 pillars distributed in the cavity of pattern 6 (P6). This design is an extension of patterns 4 and 5. The pillar geometries are similar to those in P5 (Figure 2f, Table 1). 

### 2.2. Fabrication

The proposed designs were produced using direct laser writing, facilitated by the Photonic Professional GT2 system (Nanoscribe GmbH, Eggenstein Leopoldshafen, Germany). With a center wavelength of 780 nm, a femtosecond fiber laser was used to produce the laser beam, which was focused on the objective by Galvano mirrors [19]. Focusing objectives allowed these beams to be narrowed to a small area within the (meth)acrylate-based resin (IP-S, Nanoscribe GmbH), resulting in polymerization at that precise location. The substrate used was an ITO-coated soda–lime glass (3D MF DiLL, Nanoscribe GmbH) with a thickness variation of ±60 µm, an ITO film thickness of 18 ± 5 nm, and an ITO surface resistance of 100–300 Ω. The ITO coating enhances the refractive index contrast with respect to the 2PP resin when the ITO-coated side faces the objective, ensuring accurate polymerization. IP-S was employed as the printing medium. 

IP-S is a negative photoresist without the addition of photoinitiators. Its reactive group is methacrylate, and the curing mechanism is free radical polymerization (FRP). Provided by Nanoscribe, a UV-cured IP-S sample inhibited 2.0% of the growth of L929 mouse fibroblast cells after 72 h of incubation; therefore, according to ISO10993-5 [20], UV-cured IP-S is considered noncytotoxic [21]. In order to achieve the highest resolution possible with a 25× magnification objective, the lowest slicing (0.5 m) and hatching distances (0.3 m) were used, and due to a better structure quality of overhangs, a laser power of *p* = 75% and a scanning speed of v = 90 mm/min were selected as printing parameters. 

### 2.3. Scanning Electron Microscopy (SEM) Analysis

A scanning electron microscope (SEM; Phenom Pro Desktop SEM, Thermo Fisher Scientific Phenom-World B.V., Eindhoven, The Netherlands) was used to analyze the properties of the fabricated microstructures. SEM was employed to gather high-resolution overviews and characterize the cross-sectional views of the transferred designs (P1–P6) into microstructures. The specific settings and parameters used for SEM imaging were adjusted to represent the microstructures accurately.

### 2.4. Contact Angle Measurements

The static and dynamic contact angles were determined using the sessile drop method [22,23]. Deionized water, with a surface tension of 72 mN/m at room temperature, was considered as the test liquid. In addition to being the main component of body fluids, water has a higher surface tension than most commonly used test liquids [10]. Contact angle measurements were performed with OCA20 (Dataphysics GmbH, Filderstadt, Germany). In order to control the experiment and to perform the analysis, SCA 20-U (https://www.dataphysics-instruments.com/de/produkte/oca/software/, accesed on 14 June 2023) (Dataphysics GmbH, Filderstadt, Germany) software was used. To apply the liquid, a Hamilton 500 µL microsyringe (Hamilton Germany GmbH, Gräfelfing, Germany) with a 3.25 mm diameter and a length of 60 mm was used for this study. 

### 2.5. Immersion Test

The stability of trapped air in fabricated microstructures was evaluated by full immersion of samples into the liquid. The setup included a syringe pump, tape to fix the sample to petri dishes (3M greenback tape 851, Nanoscribe GmbH, Eggenstein-Leopoldshafen, Germany), a digital optical microscope (TOMLOV, model DM201, Aledo, CA, USA), deionized water, and a PC for data acquisition and storage. The liquid entry was controlled by a pump attached to the syringe. A silicon tube was used to realize the connection of the syringe system with the petri dish. Using an in-house setup, this subsection shows the results of the imbibing of liquid under immersion tests. 

The immersion tests were carried out by means of two different methods:

i. Gradual immersion of samples in test liquid. This method considered two endpoints for each measurement: (1) 50% of total number of cavities were filled with water, and (2) test time exceeding 24 h. To ensure statistical consistency, the immersion experiments were repeated three times for each array. The first 30 min were recorded and used for detailed comparison.
ii. Direct immersion of the microstructure in a pool of liquid to mimic the actual implantation process. Each experiment was repeated three times to increase the accuracy of the results.

### 2.6. Evaluation of Wetting under a Confocal Microscope

The cavity-filling mechanism was visualized using an upright confocal microscope (LSM 880, ZEISS, Oberkochen, Germany). The setup includes a confocal microscope with a 20× immersion objective, a petri dish, and a rhodamine and water solution. In order to prepare the fluorescent dye, a solution of rhodamine B (Carl Roth GmbH, Karlsruhe, Germany) in water was prepared with a concentration of 0.01 mM. Experiments were carried out at room temperature. The samples were first fixed to the bottom of the petri dish. The fluorescent solution was then carefully poured onto the sample from the side until it completely covered the sample. A 20× immersion objective was then lowered into the solution, after which the recoding could be started. A sequence of images with a resolution of 1024 × 1024 pixels was taken in Z-stack mode, in which 64 slices were taken from top to bottom. As a final step, an image processing program (Imaris, Oxford Instruments, Oxfordshire, UK) was used to reconstruct a 3D model of multiple cross-sections and to visualize the wetting transition over the recording time.

## 3. Results

**Figure 3:**
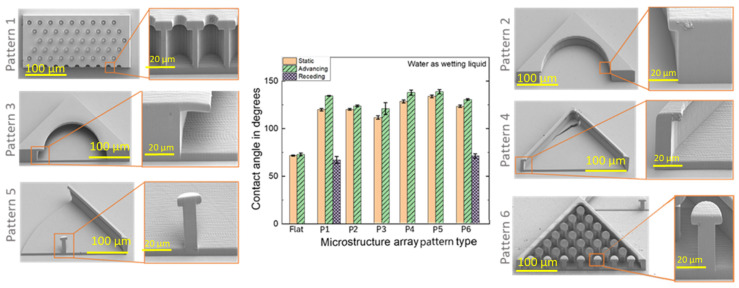
Overview of fabricated design variations and corresponding contact measurements. The provided values address advancing (green) and receding (purple) contact angles for flat and microstructure arrays with water as the test liquid (*n* = 3). Static contact angle is represented by color orange.

Fabricated samples included 100 replicates of each pattern in 10 × 10 arrays (Figure 3). The total dimensions of each printed array were 2.33 × 2.33 mm. As shown in Figure 2, only pattern 1 features 100 highly populated cavities per single block, whereas the rest are designed with a single cavity per block. It is important to note that the same cavity volume was maintained regardless of the pattern type. In the case of pattern 6, the objective was to evaluate the impact of highly populated pillars in a single cavity and investigate their impact on the non-wettability behavior of the array.

The SEM images revealed that the laser fabrication of the proposed samples was successful, and that the proposed microstructures could be printed using the selected printing parameters. However, some fabrication limitations were encountered. For instance, the dimension along the Z axis exceeded the intended size for printing in the uploaded CAD design. Furthermore, several structures were easily susceptible to fracture. This could be seen in the case of pattern 2, in which the base layer failed to be formed, or in the case of P5, with an unstable sidewall. The mechanical instability of the pillars was visible in P6, and the overhang layers in P3 were found to be tilted in some cases. For the follow-up investigations and to ensure the reproducibility of the result, only fully functional samples with minimal imperfections were used.

### 3.1. Contact Angle Measurements

Contact angle measurements were performed using deionized water. Sessile drops were applied at a size smaller than the capillary length of the liquid. As a control for all measurements, a flat IP-S layer with no microstructure was fabricated (Figure 3).

Both static (θ_0_ = 72 ± 0.6°) and advancing (θ_A_ = 73 ± 1.4°) contact angle measurements of flat samples were not fully wetted by the test liquid. This indicates the slight hydrophobic properties of the used IP-S resin. In the case of fabricated microstructures, the change in topography resulted in a much larger contact angle value compared to a flat reference surface. This was true regardless of the type of studied array. Based on the obtained data and compared to the flat reference surface, each array showed an increase in both static (111 ± 0.6° < θ_0_ < 134 ± 1.4°) and advancing (121 ± 0.6° < θ_A_ < 138 ± 2.2°) contact angles. The array pattern 4 achieved the highest static and advancing contact angles (θ_0_ = 133.6 ± 1.4°, θ_A_ = 138.5 ± 2.2°). The receding contact angle was only measurable for arrays with patterns 1 and 6 and was found to be smaller (67 ± 3.5° < θR < 71 ± 2.3°) than both the static and advancing contact angles. We observed no distinguishable receding contact angle for other patterns (2–5). This can be attributed to the specific microstructural geometry of the patterns, strong adhesive forces between the liquid and the surface, or limitations in measurement accuracy. Specifically, the intricate structures of some patterns might trap the liquid or create strong adhesive forces that prevent the formation of a clear, receding meniscus.

### 3.2. Immersion Tests

In addition to the contact angle measurement, the immersion test characterized the fabricated arrays. This allowed us to determine the nature of air entrapment and wetting in a microstructured array when immersed in a liquid. The results obtained from the two types of experimental setup are presented below.

#### 3.2.1. Evaluation of Water Imbibing in the Cavity

**Figure 4:**
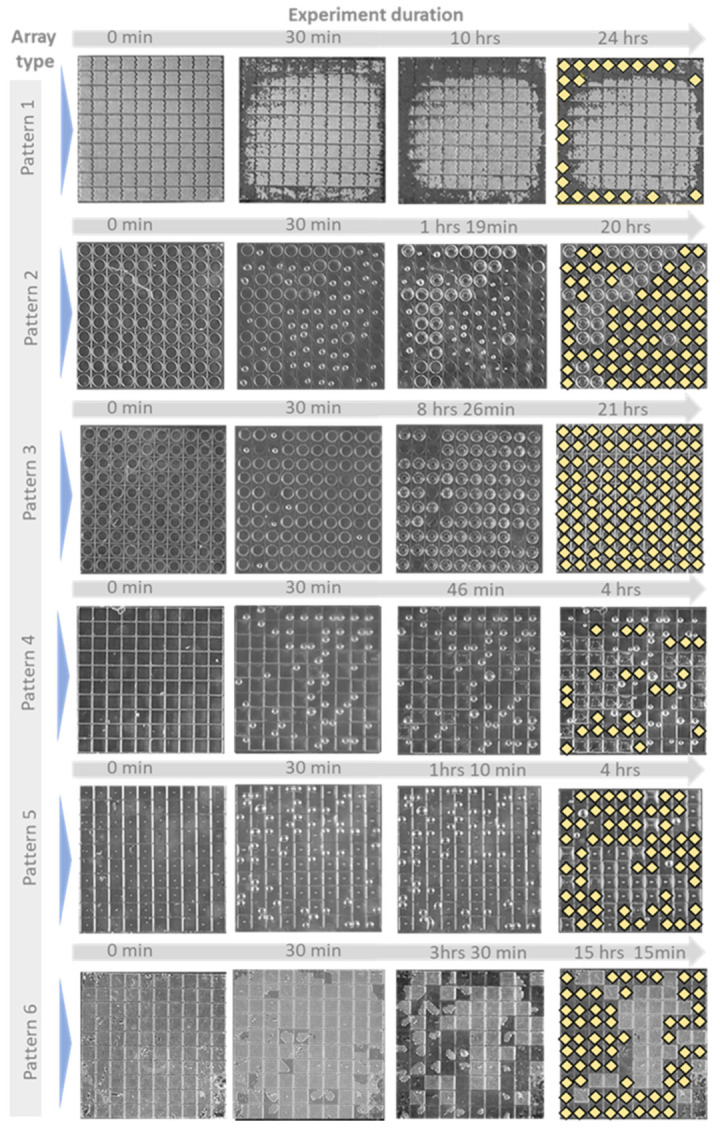
Top-view optical micrographs of patterns 1 to 6 taken at different intervals. The water entry is shown by blue arrow at the left side; completely filled cavities at the end of the experiment are indicated with yellow signs. Filled cavities often appear darker due to water’s refractive index, and the absence of reflections distinguishes them from unfilled ones.

**Figure 5:**
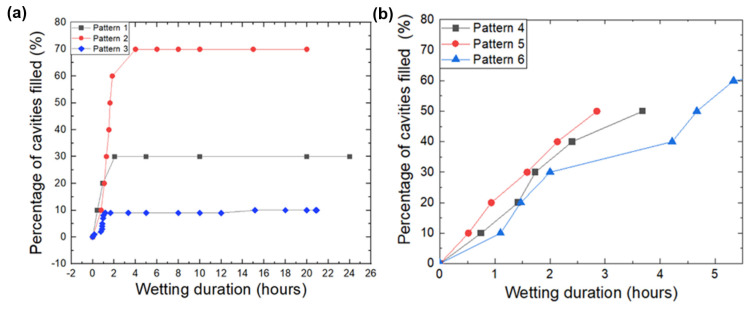
Progress of the filling percentages of cavities vs. the wetting duration for the respective design variations extracted from Figure 4, accounting for the impact of (**a**) size of the air-filled cavity (arrays with patterns 1 and 2) and length of the overhang layer (arrays with patterns 2 and 3); (**b**) the number of pillars (arrays with patterns 4, 5 and 6).

In the immersion tests, the liquid entered the cavities, which released the trapped air from the microwells (Figure 4). A sequential description of the wetting transition for microstructure arrays is provided for each design variation. For each pattern, optical micrographs are shown at different wetting intervals. The results of this experiment are summarized in Figure 5, where the percentage of the filled cavities is plotted versus the experiment duration. This highlights the influence of each pattern on the wetting behavior of the tested arrays. It was observed that water imbibing in the cavity up to 50% of the array ranged between 3 h and over 24 h after the initial contact with the test liquid (Figure 4). If half of the array was completely wetted or if the recording time exceeded 24 h, the experiment was terminated. This definition of “failing time” is consistent with the criteria outlined in existing literature [10]. In the case of patterns 2 and 6, it was decided to continue with the monitoring, as it was noticed that despite 50% being reached after 80 min, the wetting percentage remained stable over 22 h.

##### Array Pattern 1

Water imbibing in arrays with pattern 1 was observed first in cavities close to the sample edge, but they remained intact at the center. A higher level of filling was also observed in cavities close to the water inlet. After 30 min, some cavities around the center were filled as well. The structures remained intact up to 24 h after the initial immersion, with little to no further filling. The recording was stopped after 24 h, and filled cavities were manually marked and counted (Figure 4). Sixty-five structures (65% of the array) remained empty 24 h after the start of the experiment. 

##### Array Pattern 2

It was observed that the pinning of bubbles occurs instantaneously as a result of the use of a large-volume circular cavity (pattern 2). This occurred within two minutes of the water reaching some of the structures. For structures closer to the water ingress area (Figure 4), this effect seemed to be more pronounced. As the wetting duration increased, air bubbles at the center of the cavity bulged and gradually decreased in diameter. This caused the air bubble to become trapped at one side of the cavity and eventually break out of the water. Over time, the percentage of filled cavities gradually increased. Within about an hour and 37 min, more than half of the array was completely filled. The filling appeared to saturate after a period of 4 h and 25 min, after which no further bubbles were observed until the experiment was terminated after 20 h. It should be noted that the other unfilled cavities (26% of the array) also exhibited significant air bulging; however, they remained unchanged till the experiment’s end.

##### Array Pattern 3

In arrays with pattern 3, the influx of water seemed to start from the bottom of the structure. In the first eight minutes, two cavities reached the state of filling, whereas seven cavities showed the formation of air bubbles. Meanwhile, there were several cavities that showed a bulging air filling, which is the first step before bubbles finally form in the cavity. During the experiment, it was observed that it takes a considerable amount of time for a cavity to go from a bulging state to one in which an air bubble is formed. Until 20 h and 56 min, only one cavity was transiting into an air bubble. However, it was found that with an extended wetting duration, there was an increased degree of bulging for all cavities that were not filled yet. In just four minutes, all the remaining cavities were filled (Figure 4).

##### Array Pattern 4

In the case of arrays with a singular pillar design, water was imbibed into the array. Air bubbles appeared immediately at the site closest to where water entered the array. The imbibing of liquid caused the cavity at the center to bulge and form a meniscus layer on all four sides. With an increase in wetting duration, the bulging structure transformed into a bubble, which was found to be always pinned to one of the corners of the structure. There was a gradual reduction in the size of the air bubble prior to release, which was an indication that the filling process had been completed. It took 3 h and 41 min for 50% of the cavities to be filled when considering the entire array. Approximately 31% of the array remained unfilled by the end of the recording (after 4 h).

##### Array Pattern 5

In arrays with pattern 5, the influx of water occurred from the right side, resulting in the immediate wetting of several cavities and the release of air bubbles (Figure 4). The wetting process began with the bulging of air, followed by the formation of a semicircular air pocket, which formed at one side of the cavity with the help of the center pillar. 

As the duration of wetting increased, an air bubble formed at the corner, which remained pinned to the pillar. The steps that followed were similar to those observed in pattern 4. On optical micrographs, the transition of marked structures (orange dots) from a non-wetting state to a filled state in Figure 4 can be seen. In the case of arrays with pattern 5, the entire array reached 50% filling after 2 h and 51 min. 

##### Array Pattern 6

In this case, wetting started from one corner and progressed mainly along the edges before the whole structure became fully wetted. After four hours and forty minutes, 50% of the array had been filled. The array filled gradually over 9 h, and no further wetting was observed until the experience was terminated, with 31% of the array still remaining in the non-wetting state. 

Figure 5 illustrates the progression of filling percentages in cavities over time under wetting conditions, comparing different design variations. Figure 5a focuses on circular cavities, presenting the immersion results for array 1 and array 2, which differ in the size of the air-filled cavity, and array 2 and array 3, which differ in the length of the overhang layer. The results demonstrate the ability of these designs to retain air pockets stably. In contrast, Figure 5b concentrates on rectangular cavities, examining the impact of the number of pillars in array 4, array 5, and array 6. These results offer insights into the filling behavior of rectangular cavities under varying conditions.

#### 3.2.2. Mimicking the Implantation Process 

**Figure 6:**
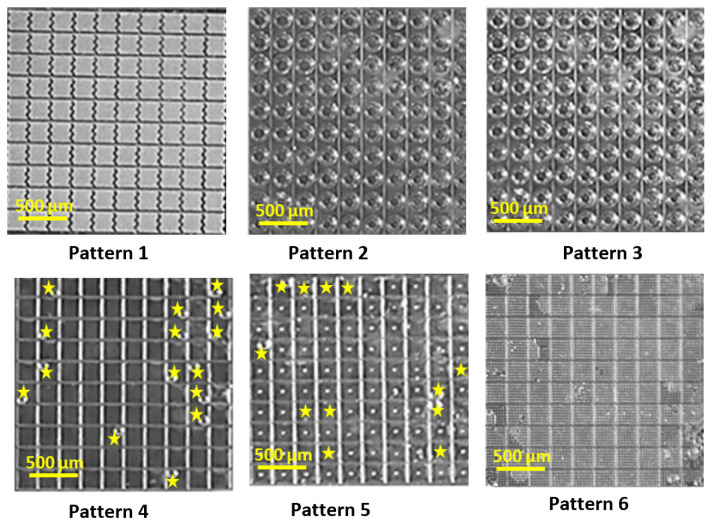
Optical micrographs (top view) of the microstructure arrays after 30 min of direct immersion in the pool of water. Filled cavities were identified by their darker appearance. Yellow stars are used to mark cavities filled at the end of the recording time.

The previously presented results highlighted the outcomes of immersion tests with a constant liquid flow, which provided a thorough understanding of the wetting behavior of the proposed microstructures. In real-life scenarios, however, the neural implants must be inserted directly into a pool of body fluid. In order to simulate an implantation process, each microstructure array was immersed directly in a pool of water for 30 min, and their wetting behavior was studied (Figure 6). 

The wetting behavior of the cavities was observed to be very similar to the results obtained in Section 3.2.1. Compared to the controlled entry of water, the percentage of fully wet areas within 30 min was found to be lower for all arrays. In the case of arrays with pattern 1, no wetting was observed within 30 min, and no single cavity reached a filled state in any of the three repetitions. For arrays with pattern 2, no cavities reached a filled state in repeated experiments, although four cavities showed signs of air bubble formation. In the case of the array with pattern 3, only one cavity was filled in the first experiment, whereas in two repetitions, no filling of the cavity was observed within 30 min. As the wetting duration increased, the air pocket bulged, although no bubbles were formed. A maximum of 15 bubbles were formed in repeated experiments for the array with pattern 4, yet no air bubbles were released. In the array with pattern 5, no cavity was completely filled within 30 min, although a maximum of 12 bubbles formed during the experiment. For all three repetitions of array pattern 6, no wetting was evident within 30 min. 

## 4. Discussion

The contact angle (CA) measurement was first utilized to measure the non-wettability properties or resistance against wetting between the fabricated surfaces. The contact angle has remained one of the most important values measured experimentally during the characterization of solids and their wetting characteristics [1,24]. Comparing the CA results (Figure 3) with the performed immersion tests (Figure 5) suggested that the CA tests did not qualify merely as a reliable measure to evaluate the differences in wettability between the pattern variations. CA measurement describes a junction between three phases and is a suitable approach for analyzing hydrophobicity and hydrophilicity for flat surfaces. However, it is not an ideal approach to discuss the wettability properties of an interface. High hydrophilicity allows water to enter more easily and increases wettability, whereas high hydrophobicity prevents water from slipping on solids, thereby increasing their non-wettability. Combining these two concepts, wetting and hydrophobicity, in the analysis of the non-flat CA test results creates many unanswered questions and introduces additional complexity to the problem. 

The receding angle is the contact angle between a liquid and a solid that has already been wetted with the liquid and is in the process of being de-wetted. Only patterns 1 and 6 had receding CAs, due to the small diameter cavities and micropillars that act as obstacles to droplet entry. This suggests a potentially weaker interaction between these patterns and the liquid as compared to others, where the receding angle could not be measured. Accordingly, patterns 2, 3, 4, and 5 show a clear influence on the interactions at the solid–liquid interface. 

Even though the presented CA measurement results do not provide information on the ability to save air pockets within the solid state, it is noteworthy that patterns 4, 5, and 6 had a greater free space to save air packets (with about 1.177 × 10^6^ µm^3^ capacity), and a higher CA value was measured for them as compared to patterns 1, 2, and 3, which had a smaller free space to save air packets (with about 7.85 × 10^5^ µm^3^ capacity). This suggests that the available free space within the microstructures might influence the overall wettability behavior, with larger spaces potentially offering better resistance to liquid penetration. 

**Figure 7:**
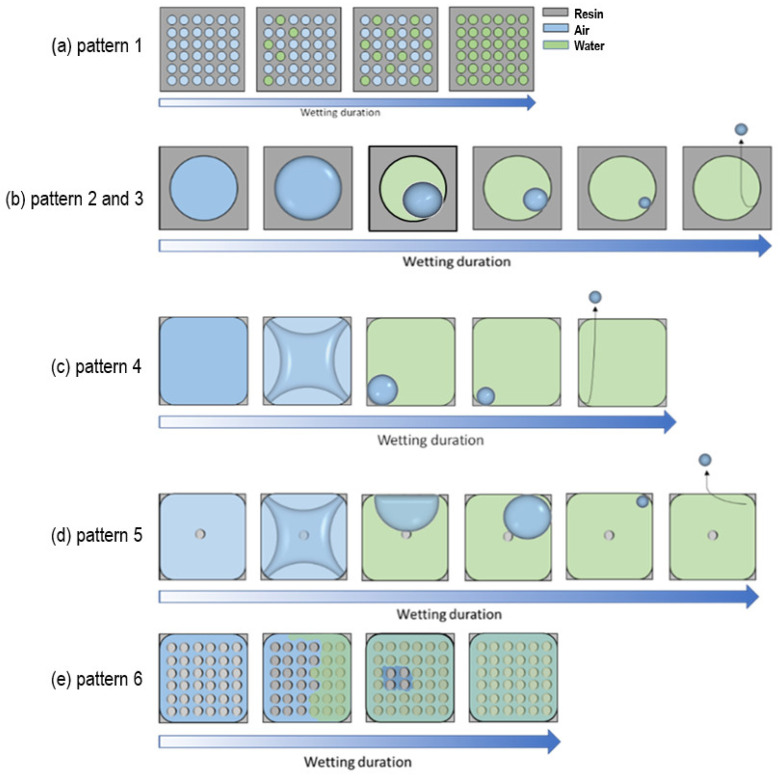
Schematic summary of the observed filling mechanisms of each pattern for an increasing wetting duration.

To further investigate the relationship between the increase in CA and the size of the gas pockets that separate the solid and liquid, all design variations were subjected to a full immersion experiment. Extracted from the results presented in Figure 4, a graphical representation summarizes the wetting behavior of each single pattern (P1–P6) in the tested arrays (Figure 7). As shown in the schematic derived from the immersion test results, the corners are preferred by water for imbibing, whereas in the absence of corners, water could not enter into the circular cavities easily. The concentration of mechanical stress at the solid–liquid–gas triple lines increases with the sharpness of the corners due to geometric stress concentration effects. This relationship can be described by the Young–Laplace equation, which relates the capillary pressure difference across a liquid interface to its curvature and surface tension [25,26]. Similarly, other groups suggested that the sharpness of the corners was proportional to the concentration of mechanical stress at the solid–liquid–gas triple lines, somewhat like the concentration of stress at the corners in solid plates under tension [22]. Due to various factors, such as mechanical vibrations or the formation of a conjoining precursor film over time, this stress concentration at the corners could lead to the liquid meniscus falling onto the inner walls [10]. After a falling time, the curvature of the liquid–vapor interface changed, and the air trapped inside the cavity began to diffuse into the liquid [10]. 

### Visualize the Cavity-Filling Mechanism

**Figure 8:**
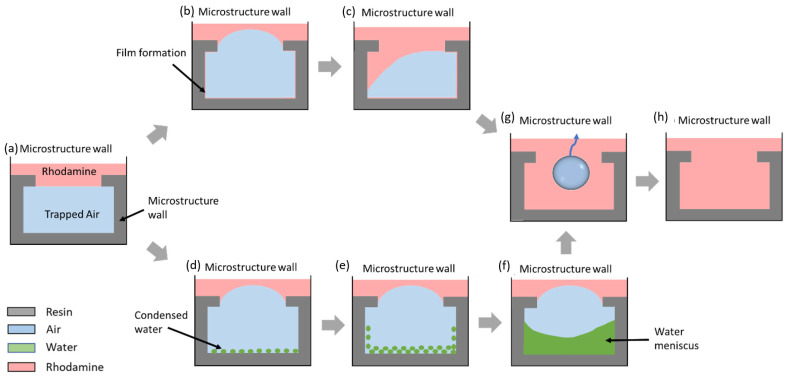
Schematic diagram summarizing the wetting mechanism investigated by upright confocal microscope (LSM 880, ZEISS, Oberkochen, Germany): (**a**) stabilized liquid meniscus at the start of the experiment, (**b**) bulging of trapped air cavity due to the formation of a thin film of wetting liquid along the edges and the walls of the reentrant structure, (**c**) pinning of air bubble due to asymmetric filling of cavity, (**d**) condensation of air into water droplets at the base of the structure, (**e**) progression of condensed monolayer into a multilayer, (**f**) formation of water meniscus due to accumulation of condensed layer, (**g**) formation and release of air bubble, (**h**) completely filled state of the microstructure.

An upright confocal microscope (LSM 880, ZEISS, Oberkochen, Germany) was used to visualize the cavity-filling mechanisms. This was applied only for the two design variations with the longest wetting transitions. This provided a detailed insight into the wetting mechanisms, including the different wetting phases at which the degassing of the cavities occurred. The general wetting behavior of reentrant microstructures that were fabricated in this work was summarized in a schematic diagram presented in Figure 8. 

**Figure 9:**
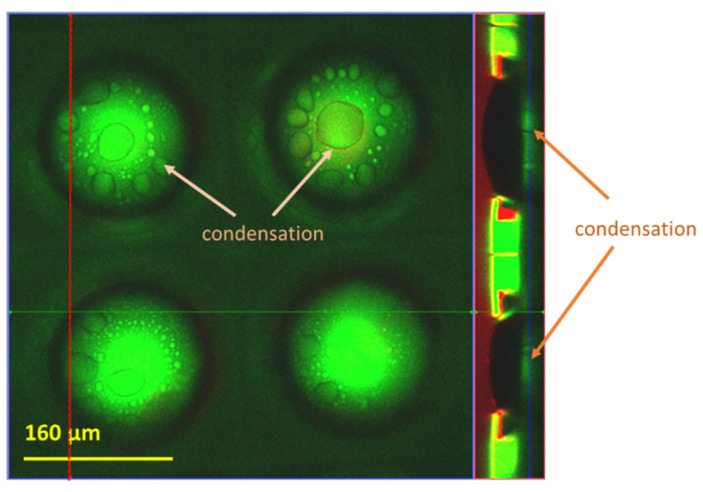
Confocal microscopy top view and cross-sectional images of array 6, showing condensation at the base of the cavities. The red region (cross-sectional view on the right) corresponds to rhodamine, green to resin, and black to air, respectively. The cross-section image is taken along the red dotted line of the top view.

At the start of the experiment, rhodamine was used to form a stabilized layer at the edge of the reentrant structure (Figure 8a), with air trapped beneath the rhodamine layer. As the wetting duration increased, the results suggest the possibility of two wetting phenomena. As described in Figure 8b, in the first case, rhodamine solution imbibes along the edges of the reentrant structure and wets the wall, thus forming a thin film of rhodamine at the base of the structure. As indicated by [10], the formation of such a thin layer in the base is likely to propel trapped air up the sides of the base and thus cause a bulged meniscus to form. This bulged meniscus was observed during optical microscopy investigations. Over time, the rhodamine solution enters the cavity, causing an asymmetrical filling of the meniscus, which causes the trapped air to pin. This pinning of trapped air frequently results in the formation of an air bubble, which is then pinned to one of the structure’s edges before being released. The capillary condensation of trapped air is another possible explanation for the wetting behavior, as shown in Figure 8d,f. Simply stated, condensation is the process by which the vapor phase changes into the liquid phase, which occurs when the vapor pressure is greater than the saturation pressure [27]. This principle, however, does not hold true when macropores are present on the surface, which is the case for most of the microstructures fabricated in this study [28]. In accordance with the Kelvin equation for macropores [29], condensation can occur when the vapor pressure is less than the saturation pressure, and for that reason, bubbles become smaller when pinned at the edges of macro cavities. The condensation of some of the cavities of array pattern 3 was inspected in detail using confocal microscopy (Figure 9). The observed filling process suggested that the condensation would gradually lead to the formation of a monolayer and eventually change to a multilayer condensed liquid at the base of the substrate. A water meniscus is also expected to form as a result of the accumulation of condensed droplets. In a dynamic process of transitioning from a monolayer into a multilayer condensed film, the trapped air cavity bulges out (Figure 8f). This was followed by the formation and, eventually, the release of the air bubble. It is also worth noting that both mechanisms can occur simultaneously in different cavities of a microstructure array. Based on the knowledge gained from the wetting progression in arrays with different morphologies and dimensions, the effect of each design feature is discussed below.

The impact of cavity distribution: As shown in Figure 5a, array pattern 1 and array pattern 2 are compared in order to evaluate the impact of increasing the number of air-filled cavities while maintaining the same total air volume. In array pattern 1, there were 100 times more cavities than in array pattern 2. Array pattern 2 reached 50% filling within 2 h, whereas array 1 only reached 30% filling within 24 h. The ratio of the open area diameter to the cavity base diameter is presented by D/d, which was smaller in the case of pattern 1 as compared to pattern 2. Accordingly, the air pocket pressure at the open site in pattern 1 is higher than the air pocket pressure at the open site in cavities in pattern 2, which can explain the difference in the time needed for wetting. In the case of small cavities, the key steps of the wetting process, i.e., imbibing liquid and forming a condensation layer beneath, could occur in each cavity independently. Hence, it should have a superior non-wetting property as compared to single-cavity arrays (pattern 2). 

Impact of overhang layers: The effect of the overhang length on the wetting behavior of each array pattern was investigated (Figure 5a), with array pattern 3 having a fourfold increase in overhang length compared to array pattern 2. The length of the overhang significantly influenced wetting behavior, with the time required to achieve 50% wetting of the array for pattern 3 being more than 13 times longer than for pattern 2. It is expected that a longer overhang will increase the time for the immersed liquid to imbibe along the edges of the reentrant structure, as well as increase the strength of the pinning of the air packets. Pattern 3 had a smaller open area diameter than pattern 2, and the air pocket pressure at the open side of the cavity was higher than the air pocket pressure in the open site; thus, the liquid had to exert more force and needed more time to enter the cavity. On the basis of the results presented, a further hypothesis was that the longer overhang facilitates the anchoring of the trapped air cavity under the reentrant structure for a longer period of time, thus prolonging the formation of the air bubble. To the authors’ knowledge, a comparison of the overhang length of a reentrant structure has not yet been performed, although several studies [10,30] comparing the wetting transition of simple and reentrant structures show a slower transition for the latter due to its overhang. This is the first time a long overhang layer microcavity has been used for non-wettable microstructures that can save air three times longer in duration than similar ones [2,22]. 

Influence of pillars: In order to investigate the role of pillars in wetting transition, array patterns 4, 5, and 6 were compared (Figure 5b), where array 6 consisted of 49-fold more pillars than array 5, and array 4 consisted of no pillars. The array with the highest number of pillars showed a longer wetting transition among the investigated arrays, suggesting that a large number of pillars could provide additional support to withstand the imbibing of liquid. In array patterns 4 and 5, air bubbles were pinned at the corners of the structure before they were released. This could be attributed to the edge effect [31] induced by the sharper corners present in these two structures, as edges and other physically induced heterogeneities are known to form so-called pinning sites [32]. In terms of the wetting behavior of microstructures, pillars have two opposing effects. It is the sidewalls of the pillar that facilitate the sliding of liquids and speed up the wetting process. Another effect is their resistance against the liquid’s entering force. Further studies are necessary to investigate the effect of the distribution, height, and position of micropillars in cavities on non-wetting behavior. 

## 5. Conclusions

Based on our findings about the impacts of different design features, the wetting transition seemed to be dependent upon (a) the size of the cavity, (b) the rate of imbibition along the edge and wall of the cavity, (c) the rate of condensation of air at the bottom and wall of the cavity, and (d) the solubility of air in water with respect to pressure applied.

Specifically, the smaller 10 μm cavities in pattern 1 led to only 30% filling after 24 h, compared to over 50% filling in 2 h for the larger 190 μm cavity in pattern 2. The highly populated small cavity arrays in pattern 1 exhibited superior non-wettability, with 65% intact after 24 h versus only 26% intact for the single large cavity in pattern 2 after 20 h. Increasing the overhang four fold from 5 μm in pattern 2 to 20 μm in pattern 3 extended the 50% wetting time from 1.6 h to over 20 h. The addition of 49 pillars in pattern 6 increased the 50% wetting time to 4.7 h, compared to 3.7 h without pillars in pattern 4. 

In order to discuss the filling rates of a test structure, it is essential to consider both surface energy and viscosity. In this study, we used DI water as the test liquid for both CA and immersion tests. Despite having a higher surface tension than the actual body fluid, our results provide comparative data on the wetting performances among different surface structures. Future studies should include more sophisticated test liquids such as CSF and plasma to discuss how differences in surface tension and viscosity can influence the presented conclusions in the paper. In this study, Nanoscribe’s IP-S was used to fabricate the microstructures. IP-S is a non-cytotoxic resin formulation that has been reported in several publications to be suitable for biological applications [33,34,35,36,37]. As a future step, it would be necessary to extend the findings of this study with a larger sample size to determine the effectiveness of the proposed surface modifications. 

Application significance: Modification of neural implant surfaces could significantly improve interactions with host tissues during the acute and chronic phases [3]. In particular, non-wettability is thought to be important in modulating the acute immune response in the first hours after injury. This is granted by the entrapped air, or potentially any target gas, which minimizes the direct surface contact between the solid state and the body fluid. This, in turn, can have an impact on the adsorption of the signaling biomarkers, which trigger an immune response cascade [8,9,38]. Furthermore, the presence of pillars on the surface of the implants is also of interest as they increase the chance of anchoring neural synapses [39].

To conclude, this work demonstrated the possibility of achieving non-wettable surfaces through tailoring topographical modifications without requiring any chemical modification of the surface structures. In the context of neural implants, this highly non-wettable surface design can offer great benefits, as it could minimize the direct interaction of neural implants with the host tissue during the first few hours after implantation. This should be of interest to the neural engineering community, as the acute implantation phase is a crucial stage that can affect the long-term success or failure of the neural device. Future in vitro, as well as in vivo studies, should further evaluate the proposed strategy in the context of neural implants.

## Figures and Tables

**Table 1:** Details of the dimensions used for microstructure patterns P1 to P6. The given dimensions are for a single cavity, D = top diameter or length of the top curve cube sides in P4–P6, d = base diameter or base curve cube side length, T = wall thickness, l = overhang length, h = overhang height, H = structure height, B_H_ = base height, C_r_ = radius of the corner curve, and N.Pillars = number of micropillars per each cavity. The empty cells in the table are explained by the fact that not all patterns featured pillars or curved opening corners.

Pattern	D (µm)	d (µm)	T (µm)	l (µm)	h (µm)	H (µm)	BH (µm)	Cr (µm)	N.Pillars
1	10	20	3	5	1	25	4	-	-
2	190	200	3	5	1	25	4	-	-
3	160	200	3	20	1	25	4	-	-
4	217	227	3	5	1	25	4	15	-
5	217	227	3	5	1	25	4	15	1
6	217	227	3	5	1	25	4	15	49

## Data Availability

This can be provided upon request.
